# Computational and Functional Characterization of Angiogenin Mutations, and Correlation with Amyotrophic Lateral Sclerosis

**DOI:** 10.1371/journal.pone.0111963

**Published:** 2014-11-05

**Authors:** Aditya K. Padhi, Kamalika Banerjee, James Gomes, Manidipa Banerjee

**Affiliations:** Kusuma School of Biological Sciences, Indian Institute of Technology Delhi, New Delhi, India; University of Florida, United States of America

## Abstract

The Angiogenin (*ANG*) gene is frequently mutated in patients suffering from the neurodegenerative disease - amyotrophic lateral sclerosis (ALS). Most of the ALS-causing mutations in Angiogenin affect either its ribonucleolytic or nuclear translocation activity. Here we report the functional characterization of two previously uncharacterized missense mutations in Angiogenin - D22G and L35P. We predict the nature of loss-of-function(s) in these mutants through our previously established Molecular Dynamics (MD) simulation extended to 100 ns, and show that the predictions are entirely validated through biochemical studies with wild-type and mutated proteins. Based on our studies, we provide a biological explanation for the loss-of-function of D22G-Angiogenin leading to ALS, and suggest that the L35P-Angiogenin mutation would probably cause ALS symptoms in individuals harboring this mutation. Our study thus highlights the strength of MD simulation-based predictions, and suggests that this method can be used for correlating mutations in Angiogenin or other effector proteins with ALS symptoms.

## Introduction

Amyotrophic Lateral Sclerosis (ALS), also known as Lou Gehrig’s disease, is a fatal progressive neurodegenerative disorder characterized by the preferential loss of motor neurons in the brain stem, motor cortex and spinal cord. As a result of this degenerative process, most patients die from paralysis and respiratory failure between 3–5 years of onset of symptoms [1]–[3]. There is no primary, disease-halting therapy available for ALS at present; the only drug for treatment being an antiglutamatergic compound, riluzole, which extends the lifespan of ALS patients briefly, albeit without any improvement of symptoms.

**Figure 1:**
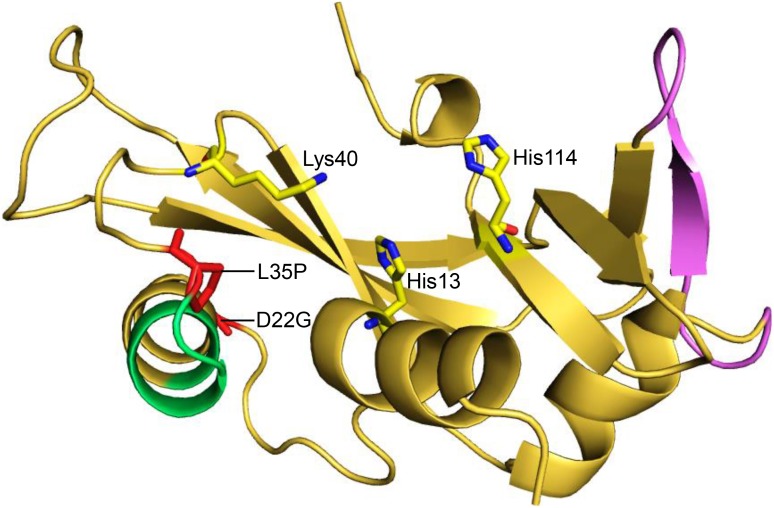
Cartoon representation of human Angiogenin showing its functional sites and D22G, L35P mutations. The D22G and L35P mutations in Angiogenin (PDB ID: 1B1I) are labeled and represented as stick models (red). The three key functional sites of human Angiogenin (catalytic triad, nuclear localization signal and receptor binding site) are also shown. The color scheme is as follows - Angiogenin protein: yellow, nuclear localization signal: green, receptor-binding site: violet and catalytic triad of Angiogenin: yellow and blue stick models.

Mutations in a number of genes have been identified as causative factors for ALS. For example, missense mutations in genes such as *SOD1*, *FUS*/*TLS*, *TARDBP*, *ANG*, *VAPB*, *DAO*, *OPTN*, *VCP*, and a recently identified hexanucleotide-repeat expansion (GGGGCC) in *C9ORF72* have been documented to be ALS causative [2]–[5]. One of the frequently mutated genes in ALS patients is *ANG*, which encodes a 14.1 kDa neuroprotective effector, Angiogenin [6]. For execution of its essential neuroprotective functions – such as neovascularization, induction of angiogenesis, stimulation of neurite outgrowth and path finding, and protection of motor neurons from hypoxia-induced death, Angiogenin utilizes three distinct functional sites [7]. The first site, a catalytic triad comprising residues His13, Lys40 and His114, is responsible for its ribonucleolytic activity; the second site, a nuclear localization signal ^29^IMRRRGL^35^, helps in nuclear translocation of Angiogenin and resultant ribosomal biogenesis, protein translation and cell proliferation; while the third site, ^60^NKNGNPHREN^68^_,_ acts as a receptor-binding site, allowing binding of angiogenin to motor neurons and endothelial cells (Figure 1). It has been shown previously that mutations which affect either the ribonucleolytic activity or the nuclear translocation activity of Angiogenin can cause ALS [8]–[10]. A total of 29 mutations in *ANG* have been identified in ALS patients so far, of which 19 missense mutations have been correlated with alterations in the functionality of Angiogenin, and consequent ALS symptoms [11], [12].

We have previously reported a Molecular Dynamics (MD) simulation methodology based on certain structural and dynamic attributes, using which the loss-of-function(s) of ALS associated Angiogenin mutations can be effectively predicted [13]–[16]. We showed that while the loss of ribonucleolytic activity of Angiogenin is possibly due to a characteristic conformational switching of the catalytic residue His114, the loss of nuclear translocation activity is due to the local folding of nuclear localization signal residues ^31^RRR^33^, resulting in reduction of solvent accessible surface area (SASA). Some of our predictions were experimentally corroborated later, which has encouraged us to validate our predictions for uncharacterized mutants of Angiogenin [17]. Here we demonstrate, side-by-side, MD simulation-based predictions for loss-of-function mutations in Angiogenin and corroborating experimental evidence. One of the mutations, “D22G”, has been detected in a large United States ALS-phenotype cohort [18]. A second mutation, “L35P”, arising through a T195C single nucleotide polymorphism (SNP), has not yet been clinically correlated with ALS [19]. Our loss-of-function results for these mutations in Angiogenin perfectly match our MD simulation-based predictions, suggesting the proposed molecular mechanism(s) obtained from simulations are accountable for loss of ribonucleolytic and nuclear translocation activities.

## Materials and Methods

### Molecular modeling and MD simulations of wild-type and mutated Angiogenin

The crystal structure of human Angiogenin (PDB: 1B1I) was used as the starting point. The heteroatoms (crystallographic waters and cofactor, CIT) were removed from the structure before simulation. The Angiogenin proteins containing D22G and L35P mutations were prepared *in silico* by replacing the target amino acid residues with the desired ones, while keeping the secondary structures intact. All simulations were performed using energy minimizations, followed by gradual heating of the system. Each system was initially minimized employing 2500 steps of steepest descent followed by 1000 steps of conjugate gradient minimization. Topology and parameter files for the protein were generated using the “ff99SB” force field [20]. All hydrogen atoms were added using the Xleap tool of AMBER 11 [21]. The system was solvated in an octahedral box of TIP3P water with ∼10 Å between the protein surface and the box boundary. The free protein was neutralized by adding Cl− counter ions for individual models. The SANDER module of AMBER 11 package was used for all the MD simulations [21]. As described in our previous report [13], [14], all the MD simulations were performed for 100 ns in isothermal isobaric ensemble (NPT). MD simulations were carried out with periodic boundary conditions and using the Berendsen temperature coupling [22]. The algorithm SHAKE was applied to fix all covalent bonds containing hydrogen atoms [23]. The particle-mesh-Ewald (PME) method was used for treating the long-range electrostatic interactions. Simulation of Angiogenin with a tag containing the Glutathione-S-Transferase (GST) and hexa-histidine moieties was carried out in AMBER 11. The initial tagged structure was predicted and generated using the Bhageerath-H and I-TASSER protein structure prediction servers [24]–[29]. The lowest energy structure obtained from these prediction servers was used for a 50 ns long MD simulation. MD simulations were performed on a single graphics processing unit (GPU) card, installed at the Supercomputing Facility (http://www.scfbio-iitd.res.in/) of Indian Institute of Technology Delhi.

### Cloning, expression and purification of wild-type and mutated Angiogenin

The cDNA for human *ANG* gene (369 bp) was amplified by PCR from the plasmid pCMV6-XL4 (OriGene) and eventually cloned in the BamHI and EcoRI restriction sites of the *E. coli* expression vector pGEX-6P-2 (GE-Healthcare), with a C-terminal hexa-histidine His-tag. The point mutations -D22G and L35P- were generated in *ANG* through site directed mutagenesis (Stratagene). Protein expression was induced in *E. coli* BL21 cells by addition of 1 mM Isopropyl β-D-1-thiogalactopyranoside (IPTG) (Sigma), at an A_600_ of 0.6. Cells were harvested 4 hours post-induction, and cell pellets were disrupted by the addition of lysozyme (Sigma), followed by sonication (Branson sonifier 250, Netherlands). Following centrifugation at 10,000 rpm for 1 hour, the Angiogenin-GST fusion proteins were recovered in the soluble fraction. Purification of wild-type and mutated proteins was performed through standard Ni-nitrilotriacetic acid (Ni-NTA) affinity-based purification procedure (Qiagen). Fractions containing eluted fusion proteins were pooled, dialyzed and concentrated, before being further purified through size-exclusion chromatography using a Superdex 75 10/300 GL column (GE Healthcare). The concentration of wild-type and mutant proteins was determined using an extinction coefficient of 54945 M^−1 ^cm^−1^ at 280 nm (http://web.expasy.org/protparam/).

### Ribonucleolytic activity of wild-type and mutated Angiogenin

The ribonucleolytic activity of purified wild-type and mutated proteins were examined using yeast transfer RNA (tRNA) as substrate. 0.05–0.5 mg/ml of wild-type and mutant Angiogenin-GST proteins were added to a 300 µl assay mixture containing 0.6 mg yeast tRNA (Sigma), 30 µg RNase-free bovine serum albumin (Sigma), 30 mM 4-(2-hydroxyethyl)-1-piperazineethanesulfonic acid (HEPES), pH 6.8 (MP Biomedicals), and 30 mM sodium chloride (Sigma), and the assay mixtures were incubated for 2 hours at 37°C. Subsequently, 700 µl of 3.4% pre-chilled, ice-cold perchloric acid (Sigma) was added to assay mixtures to terminate the reactions. The mixtures were then rigorously vortexed, incubated on ice for 15 minutes, followed by centrifugation at 14000 rpm for 10 minutes at 4°C. The supernatants were collected, and their absorbance measured at 260 nm. The percentage loss of ribonucleolytic activity was calculated by considering the amount of mutated proteins required to generate 1.0 optical density (OD), keeping wild-type Angiogenin-GST as reference. RNAse-free water (Gibco) was used for preparing the buffers and assay mixtures to ensure RNAse-free systems. Experiments were done in triplicates.

### Circular Dichroic spectroscopy

Circular dichroism (CD) spectroscopic studies of wild-type and mutated proteins were performed on a Peltier-attached JASCO J-815 spectropolarimeter (Tokyo, Japan). Purified proteins were diluted in Phosphate Buffered Saline (PBS) to yield a concentration of 0.4 mg/ml, and measurements were taken in a 0.2 cm path-length quartz cell at a scan speed of 50 nm/min. For each sample, three spectra were recorded, averaged and plotted after subtracting the buffer baseline. Experiments were done in triplicates.

### Nuclear translocation assay

3×10^5^ HeLa cells, grown in Dulbecco's modified Eagle's medium (Gibco) supplemented with 10% Fetal Bovine Serum (FBS) (Gibco) and 1% Penicillin-Streptomycin solution (Gibco), were seeded into the central cavity of a 35 mm cell imaging dish (Eppendorf), and incubated with 2 µg/ml of wild-type or mutated proteins (D22G and L35P) for 30 minutes at 37°C. Cells were fixed with 2% paraformaldehyde (Sigma) for 10 minutes at room temperature, followed by permeabilization with 0.2% Triton X-100 (Sigma). Cells were incubated in 10% normal goat serum to prevent non-specific binding, followed by treatment with a mouse anti-Angiogenin monoclonal antibody (Abcam), at a concentration of 2.5 µg/ml for 6 hours, and Alexa Fluor 555 goat anti-mouse IgG (H+L) (Molecular Probes) for 1 hour at room temperature. The nuclei were counter-stained with 300 nM 4′,6-diamidino-2-phenylindole (DAPI) dihydrochloride (Molecular Probes). Cells were mounted in 50 mg/ml poly-vinyl alcohol (Sigma) and visualized using a Confocal Laser Scanning Biological Microscope (Olympus FV1000) through a 20X objective. Experiments were done in duplicates.

### Statistical analyses

Data are presented as mean ± the standard deviation. The differences between wild-type and mutants were determined by Student’s *t*-test (two-tailed). Significant differences were observed with p<0.05, n  = 3.

## Results

**Figure 2:**
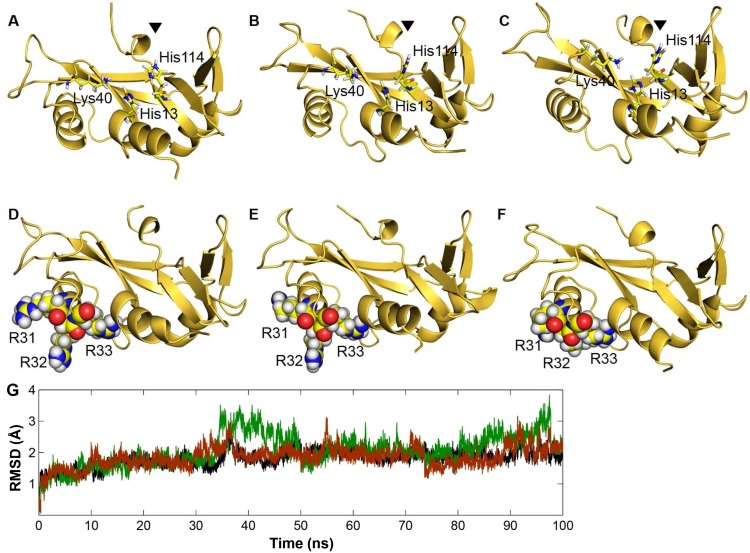
Snapshots extracted from MD simulations for D22G and L35P Angiogenin mutants. **A)** Stable and native conformation of catalytic residue His114 from MD simulations of wild-type Angiogenin **B)** Conformational switching of His114 observed from the MD simulation of ALS associated D22G-Angiogenin mutant **C)** L35P-Angiogenin mutant showing conformational switching of His114 by 99° during the simulations. **D)** Wild-type Angiogenin showing an open packing and loose conformation of nuclear localization signal residues ^31^RRR^33^ from MD simulations **E)** D22G mutant shows a similar conformation of ^31^RRR^33^ residues compared to wild-type Angiogenin, suggesting no loss of nuclear translocation activity, while **F)** a closed and tight packing of ^31^RRR^33^ residues observed from the MD simulation of L35P-Angiogenin, suggesting loss of nuclear translocation activity **G)** Control plots showing the RMSD profiles of the backbone atoms from the equilibrated conformation for wild-type Angiogenin and D22G, L35P-Angiogenin mutants during the MD simulations. RMSD time profiles for wild-type Angiogenin, D22G and L35P are shown in black, green and brown respectively.

The objective of the present study was to verify, using functional assay experiments, whether certain structural and dynamic attributes of Angiogenin mutants obtained from MD simulations result in the loss of ribonucleolytic and nuclear translocation activities. Our previous MD simulation results showed that both D22G and L35P Angiogenin mutants exhibit a characteristic conformational switching of the catalytic residue His114 by 99° from its native position [14]. We extended our previous MD simulations to 100 ns, and found that both mutants retained the His114 conformational switching during the longer simulations as well (Figures 2A–2C), while wild-type Angiogenin did not demonstrate any alteration within the catalytic triad. The HA-CA-CB-CG dihedral angle of His114 was measured during the course of simulations and it was observed that the dihedral angle changed from the mean −80° position of the wild-type Angiogenin and assumed a new dihedral orientation of −179° for D22G and L35P mutants [13], [14]. Of the two mutants, the conformational switching of His114 was most severe in the D22G mutant. Our previous results using molecular docking experiments have indicated that this conformational switching of His114 is probably responsible for the loss of ribonucleolytic activity of Angiogenin. In the altered conformation, His114 is unable to form hydrogen bond interaction and salt-bridge with NCI-65828 (a known inhibitor of the ribonucleolytic activity of Angiogenin), while in the native conformation, a strong interaction with the inhibitor results in higher binding energy score [13], [30]. Thus, based on the MD simulations of the D22G and L35P Angiogenin mutants, we predicted that both will demonstrate loss of ribonucleolytic activity, with the loss being more severe in the D22G mutant.

Further, our 100 ns MD simulation showed dynamic alteration in residues ^31^RRR^33^ of the L35P mutant. Due to local folding, this region became less accessible to solvent in the L35P mutant, resulting in reduction of SASA [12]–[15] (Figures 2D–2F). Wild-type Angiogenin and the D22G mutant retained an open conformation and loose packing of ^31^RRR^33^ residues during the simulations. Since the three successive Arginine residues (31–33) are known to play a pivotal role in the nuclear translocation activity of Angiogenin [31], [32], we predicted that a decrease in SASA in this region will result in a loss of nuclear translocation activity of the L35P mutant.

**Figure 3:**
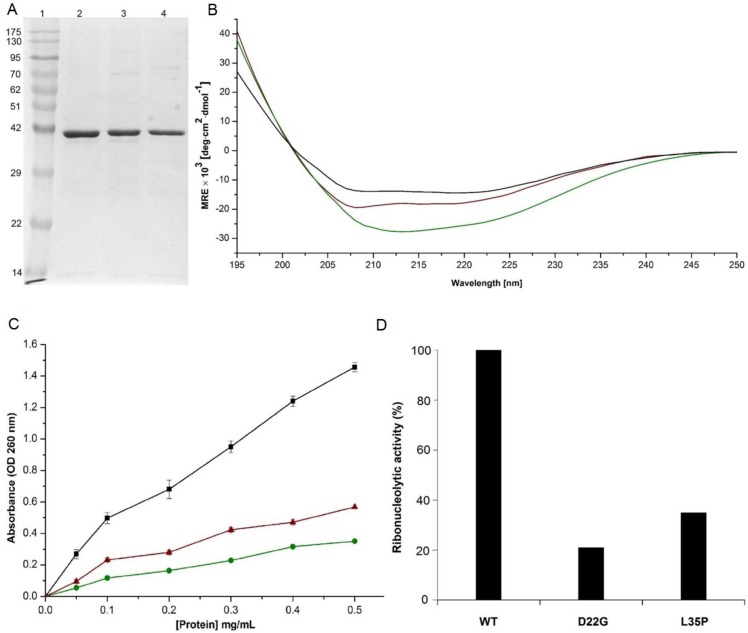
Purification, secondary structure depiction and ribonucleolytic activity of wild-type Angiogenin-GST and mutants. **A)** Coomassie stained SDS-PAGE gel showing wild-type Angiogenin-GST (lane 2), D22G (lane 3) and L35P (lane 4) proteins after Ni-NTA affinity purification. Lane 1 contains molecular weight markers. The Angiogenin proteins (14.1 kDa) are all tagged with Glutathione S-transferase (GST, ∼26 kDa) for soluble expression in *E. coli*. **B)** Plot showing CD spectra for wild-type (black), D22G (green), and L35P (brown) Angiogenin-GST proteins. Samples were diluted in PBS to yield a concentration of 0.4 mg/ml; three spectra were recorded, averaged and plotted after subtracting the buffer baseline for each sample. **C)** Ribonucleolytic activity of wild-type (black), D22G (green) and L35P (red) Angiogenin-GST proteins measured using yeast tRNA as substrate. The proteins, at concentrations of 0.05 to 0.5 mg/ml, were incubated with yeast tRNA (2 mg/ml) at 37°C for 2 hours. Undigested tRNA was precipitated by addition of ice-cold perchloric acid, and the absorbance of the supernatents was measured at 260 nm; data were collected from three independent experiments for each protein concentration. Student’s *t*-test of three independent experiments shows that the difference between wild-type and each of the three mutant protein is significant (n = 3; p<0.05). **D)** The loss of ribonucleolytic activity of D22G and L35P mutants compared to wild-type Angiogenin-GST. The amount of protein required to generate 1.0 optical density (OD) is compared with wild-type Angiogenin-GST to generate same OD unit for mutants.

In order to confirm our predictions, we generated GST-tagged constructs of wild-type Angiogenin, D22G and L35P mutants, with C-terminus His-tags. While the GST tag was required to generate Angiogenin in the soluble form in a bacterial expression system, the His-tags were included for ease of purification. The proteins were purified to >95% homogeneity through Ni-NTA affinity and size exclusion chromatography (Figure 3A), where they eluted as dimers with approximate molecular weights of 80 kDa (see Figure S1). Initial characterization of the purified proteins was carried out through circular dichroic spectroscopy. It was observed that the far-UV CD spectra of the mutants were largely similar to that of wild-type Angiogenin-GST (Figure 3B), indicating that the missense mutations did not significantly affect the secondary structure, overall stability or folding of Angiogenin-GST. The root mean square deviation (RMSD) profiles for D22G and L35P-Angiogenin also support our CD measurements, suggesting that the structural stability of the mutants was similar to that of wild-type Angiogenin (Figures 2G and 3B). In addition, MD simulation was carried out for wild-type Angiogenin conjugated with GST and a hexa-histidine His-tag, and it was observed that for the 50 ns duration of simulation, presence of the GST moiety did not affect the structural integrity or folding of Angiogenin, including its functional sites – the catalytic triad and nuclear localization signal residues (see Figure S2). This indicated that the presence of GST- or His-tag will probably not interfere with the biochemical activity of Angiogenin.

We first confirmed that the wild-type Angiogenin-GST is fully functional by comparing its ribonucleolytic activity with that of commercially available Ribonuclease A (Qiagen) (see Figure S3). Subsequently, any loss-of-function in the mutants was established through comparison of their ribonucleolytic and nuclear translocation activities with that of the GST-tagged wild-type Angiogenin. For detecting ribonucleolytic activity, equivalent amounts of wild-type and mutated Angiogenin proteins were added to a yeast tRNA substrate, the undigested tRNA precipitated by perchloric acid, and the acid soluble fragments quantified through spectroscopy. Our analyses showed that D22G and L35P-Angiogenin-GST proteins displayed 21% and 35% of the ribonucleolytic activity respectively as compared to wild-type Angiogenin-GST (considered 100%) (Figures 3C–3D). Free GST, purified similarly, and utilized in the same assay, did not show any ribonucleolytic activity. Thus, our experimental results were found to be in agreement with predictions from MD simulations, which showed that although residues D22 and L35 were located distal from the catalytic triad, mutations in these residues could potentially perturb the ribonucleolytic activity of Angiogenin. Since it is established that loss of ribonucleolytic activity in Angiogenin results in abolishment of angiogenic activity, neurite outgrowth, pathfinding and several other crucial biological functions [8], [9], it is therefore expected that D22G and L35P mutations in Angiogenin will possibly cause ALS symptoms.

**Figure 4:**
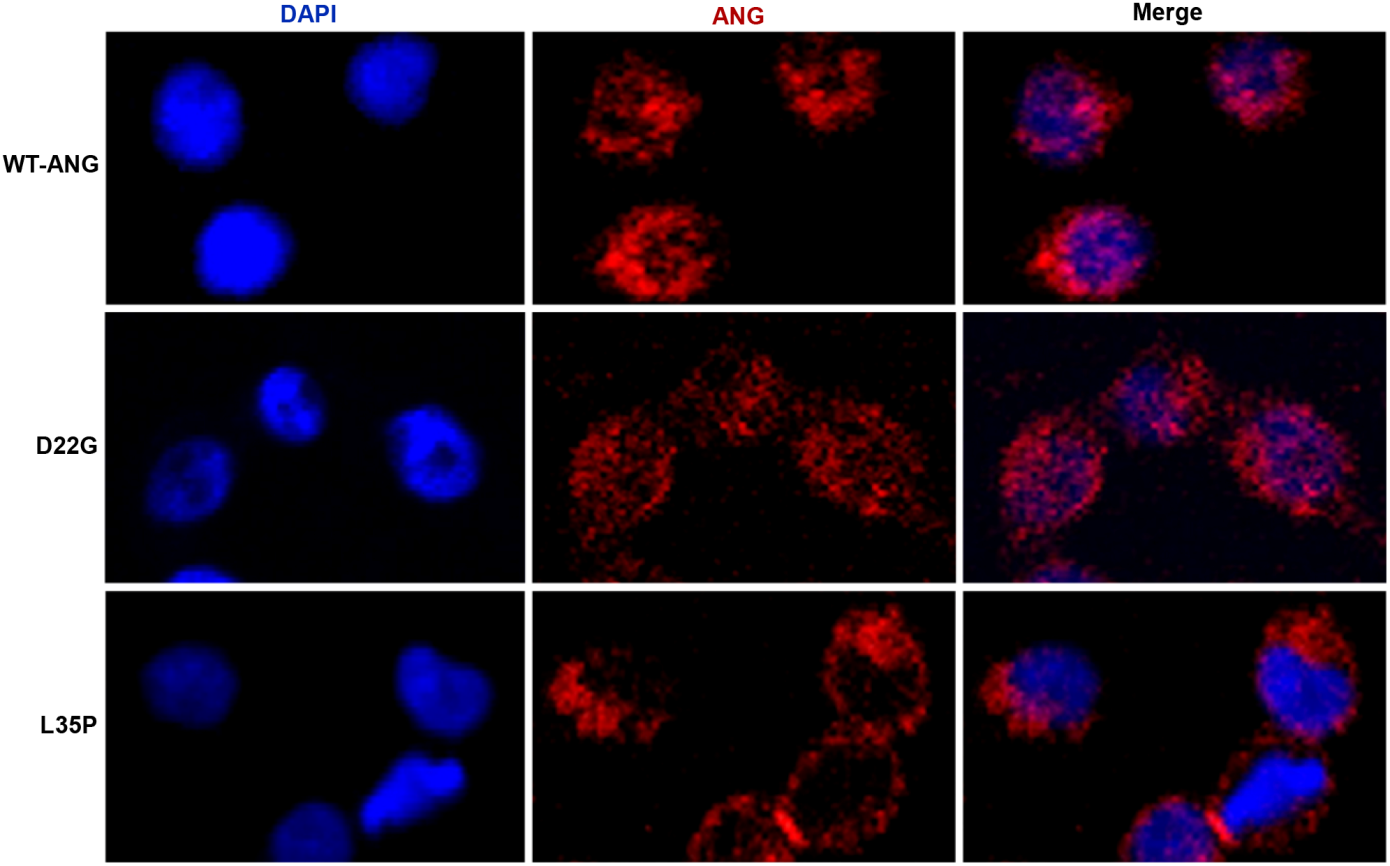
Nuclear translocation activity of wild-type Angiogenin-GST and mutants. HeLa cells, untreated or incubated with 2 µg/ml of wild-type Angiogenin-GST, or the D22G, L35P mutants were fixed, permeabilized and stained with mouse anti-Angiogenin monoclonal antibody and Alexa Fluor 555 goat anti-mouse IgG, while the nuclei were counter-stained with 4′,6-diamidino-2-phenylindole (DAPI) dihydrochloride. Individual channels, as well as a merge, are shown. The magnification in all cases is 200X. The images are representative of cells from at least three areas (each area containing 35–50 cells) from two independent experiments.

The nuclear translocation abilities of D22G and L35P mutants were investigated through immunofluorescence assays. 2 µg/ml each of wild-type and mutated Angiogenin proteins were added to cultured HeLa cells. The fixed and permeabilized cells were stained with an anti-Angiogenin monoclonal antibody, coupled with an Alexa Fluor 555 goat anti-mouse IgG labeled secondary antibody, while the nuclei were counter-stained with 4′,6-diamidino-2-phenylindole (DAPI) dihydrochloride. Untreated HeLa cells showed negligible staining for Angiogenin (see Figure S4), indicating that minor quantities of endogenous protein is produced by the cells, and the immunofluorescence detected in treated cells corresponds primarily to the extraneously added Angiogenin protein. We observed that wild-type and D22G mutant distinctly translocated into the nucleus, while the L35P mutant remained primarily in the cytoplasm, and almost no localization to the nucleus was detected (Figure 4). Thus, the results of the nuclear translocation assays were also consistant with predictions from our MD simulations, where conversion of L35 to P35 in Angiogenin resulted in the local folding of the nuclear localization signal residues ^31^RRR^33^ and consequent reduction of SASA, indicating potential abrogation of nuclear translocation.

Previous studies have shown that in the nuclear localization signal ^29^IMRRRGL^35^, the three consecutive Arginine residues ^31^RRR^33^ are critical in governing the nuclear translocation activity of Angiogenin [31], [32]. In contrast to the L35P Angiogenin mutant, the D22G mutant was able to translocate into the nucleus as effectively as wild-type. Our simulation studies for D22G-Angiogenin clearly predicted no effect of this mutation on the conformation of the nuclear localization signal in Angiogenin.

## Discussion

Molecular Dynamics (MD) simulation has been effectively utilized in addressing essential questions in basic and applied sciences. Simulation routines to probe ligand docking and protein conformational change have found valuable applications in the biomedical field for development of new inhibitors, drugs and vaccines. Here we suggest that this technique can also be successfully employed in predicting the probability of disease causation by investigating functional changes in well-characterized, disease-associated proteins, as a result of mutations detected in corresponding genes.

In this study, we have functionally categorized, through MD simulations and biochemical assays, two previously uncharacterized mutants of Angiogenin. Since its discovery, the *ANG* gene, which codes for the neuroprotector Angiogenin, has emerged as one of the frequently mutated genes found in ALS patients [6], [7]. It is well established in literature that there is a strong correlation between decreased ribonucleolytic or nuclear translocation activity in Angiogenin due to mutations, and ALS pathogenesis [6]–[8]. In order to validate our MD simulation-based loss-of-function predictions in D22G and L35P Angiogenin, we generated wild-type and mutated Angiogenin as GST fusions in *E. coli*, since free Angiogenin localizes in inclusion bodies during bacterial expression [17]. We used purified GST-tagged proteins in all assays since wild-type Angiogenin-GST had ribonucleolytic activity similar to that exhibited by commercially available Ribonuclease A (see Figure S3) and demonstrated substantial nuclear localization in HeLa cells (Figure 4). We found that the nature of loss-of-function of the two mutants, as predicted by our MD simulation routine, is entirely corroborated by functional assays. The D22G mutant had significantly reduced ribonucleolytic activity compared to wild-type Angiogenin, as this mutant exhibited a characteristic conformational switching of catalytic residue His114 during simulation (Figures 2B, 3C–3D). The observation that only the catalytic residue His114 exhibited a conformational switching was interesting, because specific mutation of this residue was previously shown to cause a 3300-fold reduction in the ribonucleolytic activity of Angiogenin, while mutating other residues in the catalytic triad caused only 300-fold reduction in activity [33]. Thus, the ALS manifestation seen in patients harboring the D22G mutation in Angiogenin is presumably due to the reduction in ribonucleolytic activity. The L35P mutant also displayed the characteristic conformational switching of His114 during MD simulation, and the predicted loss of ribonucleolytic activity was confirmed experimentally. In both the D22G and L35P mutants, a conserved hydrogen bond interaction path exists from the sites of mutation to His114, mediated through Leu115 and Thr44/Leu115 respectively [13], [14], which is possibly a valid reason for conformational switching of His114.

In contrast to the D22G mutant, however, the L35P mutant failed to localize in the nucleus of HeLa cells (Figure 4). This loss of nuclear translocation activity for L35P Angiogenin was also foretold by our simulation, based on the local folding of the nuclear localization signal residues ^31^RRR^33^ and consequent reduction of SASA (Figures 2D–2F). The L35P mutation, which is encoded by the T195C SNP, has been discovered in *ANG* but has not been clinically correlated with ALS so far. However, our finding for the L35P mutant is supported by data from publicly available resources. The F-SNP database [34], [35], which provides an integrated information about the functional effects of SNPs obtained from 16 bioinformatics tools and databases, classifies the T195C SNP, encoding the L35P mutation, as a deleterious mutation. The functional significance (FS) score for this SNP is 0.764; which indicates that this mutation might have a detrimental effect on the functionality of Angiogenin and is likely to cause ALS. Therefore, based on our simulation studies and functional analyses, we suggest that the occurrence of this SNP in the *ANG* gene is likely to result in manifestation of ALS symptoms in individuals. However, further genomic scans of ALS patients across diverse ethnic groups are required to map the association of the L35P mutation with ALS pathogenesis.

In conclusion, the strong correlation between the MD simulation based loss-of-function predictions and the functional substantiation data for Angiogenin mutants highlights the strength of the MD simulation methodology and the prediction of molecular attributes responsible for loss of ribonucleolytic and nuclear translocation activities. Therefore, this report, in addition to our earlier studies, provides a promising methodology and validation platform for understanding the possible molecular origins of functional loss of newly identified Angiogenin mutations and their association with ALS pathophysiology.

## Supporting Information

Figure S1 **Size exclusion chromatograms of wild-type Angiogenin and mutants.** Size exclusion profiles of wild-type Angiogenin (black line), and D22G (red line) and L35P (blue line) mutants, in a Superdex 75 10/300 GL column, monitored by absorbance measurement at 280 nm. All proteins eluted in peak 1, with a dimeric molecular weight of ∼80 kDa. A Western blot of each eluted fraction (peaks 1, 2 and 3) for wild-type Angiogenin-GST, carried out with an anti-His primary antibody, is shown in the right panel.
(TIF)

Figure S2 **Snapshot of wild-type Angiogenin in fusion with a GST- and His-tag extracted from MD simulation.** The Angiogenin-GST fusion protein at the end of a 50 ns simulation, with the angiogenin moiety in green and GST in orange. The catalytic triad (His13, Lys40 and His114) and the nuclear localization signal of Angiogenin did not exhibit any conformational alterations during the simulation, and maintained their native wild-type orientation. Neither the GST-moiety nor the His-tag affected the folding or structure of the Angiogenin moiety in Angiogenin-GST.
(TIF)

Figure S3 **Ribonucleolytic activity of Ribonuclease A and wild-type Angiogenin-GST proteins.** Ribonucleolytic activities of Ribonuclease A (Orange line) and wild-type Angiogenin with GST- and His-tag (Grey) measured using yeast tRNA as substrate. The proteins were used at concentrations of 0.05–0.5 mg/ml. Data were collected from three independent experiments for each protein concentration. Student’s *t*-test of three independent experiments show that the difference between wild-type Angiogenin-GST and Ribonuclease A is significant (n = 3; p<0.05).
(TIF)

Figure S4 **Detection of endogenous Angiogenin in HeLa cells.** HeLa cells were fixed, permeabilized and stained with mouse anti-Angiogenin monoclonal antibody and Alexa Fluor 555 goat anti-mouse IgG, while the nuclei were counter-stained with 4′,6-diamidino-2-phenylindole (DAPI) dihydrochloride. Negligible staining for Angiogenin in the HeLa cells indicates that minor quantities of endogenous protein is produced by the cells, and the immunofluorescence detected in Figure 4 corresponds primarily to extraneously added Angiogenin protein. The magnification in all cases is 200X. The images are representative of cells from at least three areas (each area containing 35–50 cells) from two independent experiments.
(TIF)
