# Supplementary figures and images for: Computational and Functional Characterization of Angiogenin Mutations, and Correlation with Amyotrophic Lateral Sclerosis
Source: PLoS One. 2014 Nov 5;9(11):e111963. doi: 10.1371/journal.pone.0111963 (PMC4221194; doi:10.1371/journal.pone.0111963)

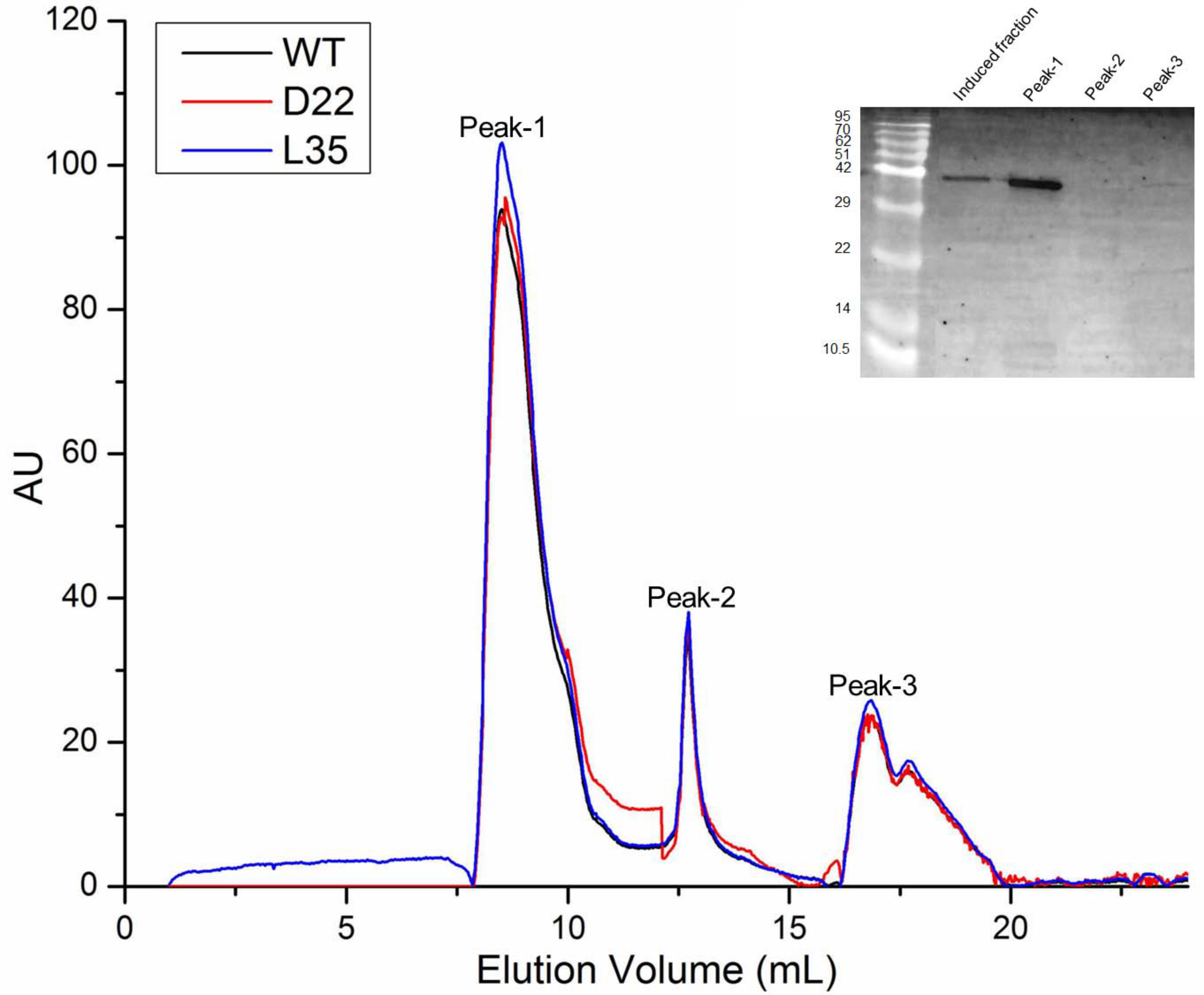

Supplement: Figure S1 — Size exclusion chromatograms of wild-type Angiogenin and mutants. Size exclusion profiles of wild-type Angiogenin (black line), and D22G (red line) and L35P (blue line) mutants, in a Superdex 75 10/300 GL column, monitored by absorbance measurement at 280 nm. All proteins eluted in peak 1, with a dimeric molecular weight of ∼80 kDa. A Western blot of each eluted fraction (peaks 1, 2 and 3) for wild-type Angiogenin-GST, carried out with an anti-His primary antibody, is shown in the right panel. (TIF) [file pone.0111963.s001.tif]

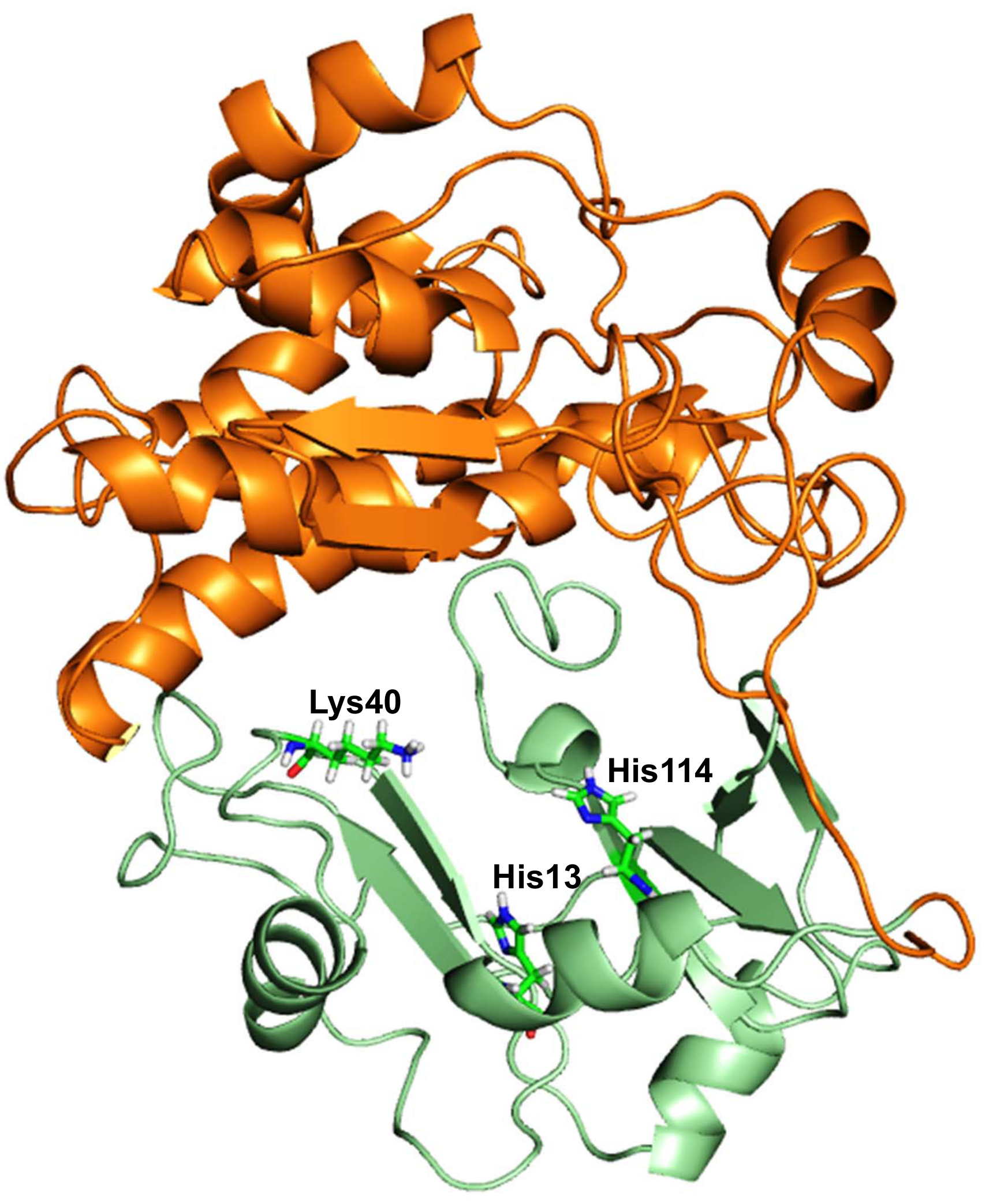

Supplement: Figure S2 — Snapshot of wild-type Angiogenin in fusion with a GST- and His-tag extracted from MD simulation. The Angiogenin-GST fusion protein at the end of a 50 ns simulation, with the angiogenin moiety in green and GST in orange. The catalytic triad (His13, Lys40 and His114) and the nuclear localization signal of Angiogenin did not exhibit any conformational alterations during the simulation, and maintained their native wild-type orientation. Neither the GST-moiety nor the His-tag affected the folding or structure of the Angiogenin moiety in Angiogenin-GST. (TIF) [file pone.0111963.s002.tif]

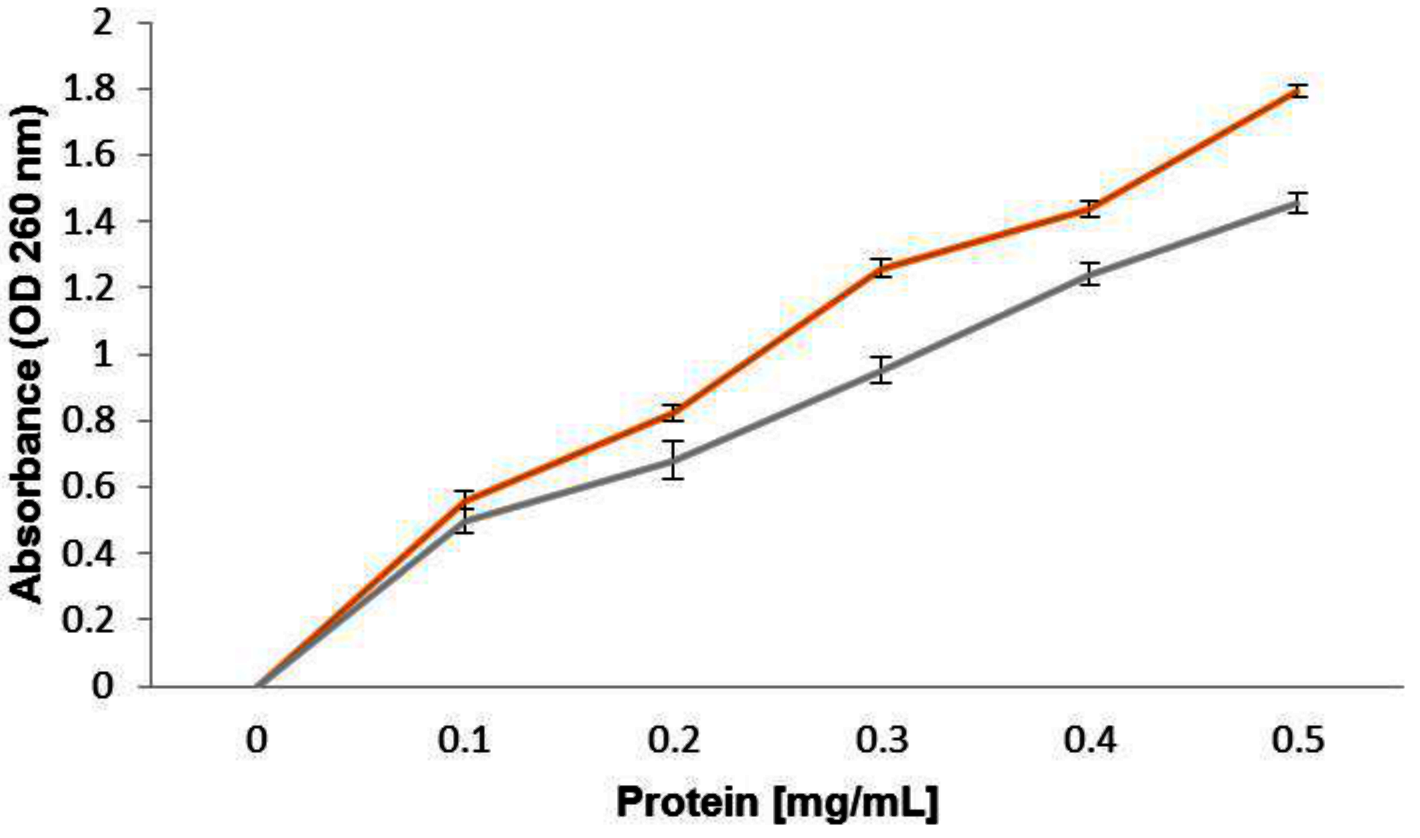

Supplement: Figure S3 — Ribonucleolytic activity of Ribonuclease A and wild-type Angiogenin-GST proteins. Ribonucleolytic activities of Ribonuclease A (Orange line) and wild-type Angiogenin with GST- and His-tag (Grey) measured using yeast tRNA as substrate. The proteins were used at concentrations of 0.05–0.5 mg/ml. Data were collected from three independent experiments for each protein concentration. Student’s t-test of three independent experiments show that the difference between wild-type Angiogenin-GST and Ribonuclease A is significant (n = 3; p<0.05). (TIF) [file pone.0111963.s003.tif]

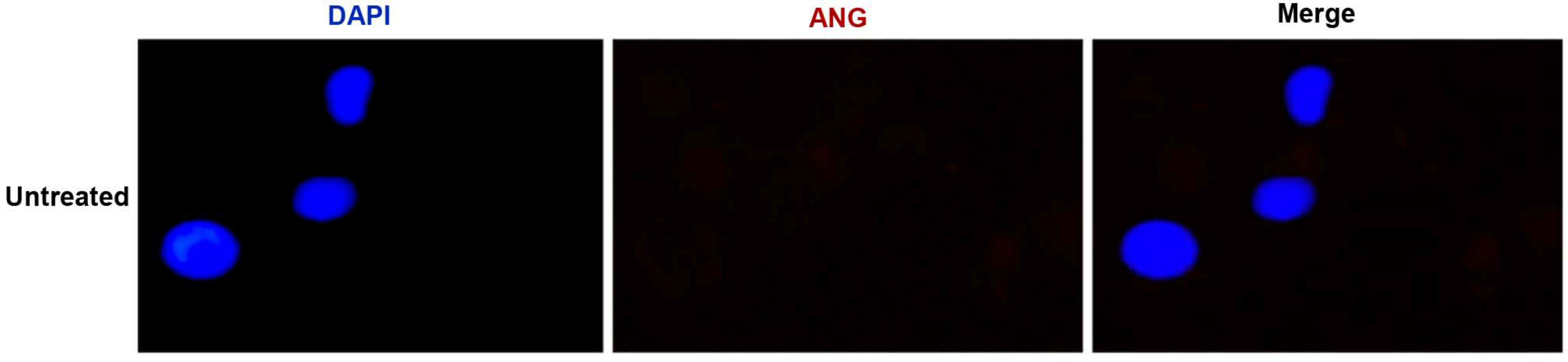

Supplement: Figure S4 — Detection of endogenous Angiogenin in HeLa cells. HeLa cells were fixed, permeabilized and stained with mouse anti-Angiogenin monoclonal antibody and Alexa Fluor 555 goat anti-mouse IgG, while the nuclei were counter-stained with 4′,6-diamidino-2-phenylindole (DAPI) dihydrochloride. Negligible staining for Angiogenin in the HeLa cells indicates that minor quantities of endogenous protein is produced by the cells, and the immunofluorescence detected in Figure 4 corresponds primarily to extraneously added Angiogenin protein. The magnification in all cases is 200X. The images are representative of cells from at least three areas (each area containing 35–50 cells) from two independent experiments. (TIF) [file pone.0111963.s004.tif]
